# Number of Cigarettes Smoked Per Day, Smoking Index, and Intracranial Aneurysm Rupture: A Case–Control Study

**DOI:** 10.3389/fneur.2018.00380

**Published:** 2018-05-31

**Authors:** Xin Feng, Zenghui Qian, Baorui Zhang, Erkang Guo, Luyao Wang, Peng Liu, Xiaolong Wen, Wenjuan Xu, Chuhan Jiang, Youxiang Li, Zhongxue Wu, Aihua Liu

**Affiliations:** ^1^Beijing Neurosurgical Institute, Capital Medical University, Beijing, China; ^2^Department of Interventional Neuroradiology, Beijing Tiantan Hospital, Capital Medical University, Beijing, China

**Keywords:** intracranial aneurysm, case–control study, risk factors, rupture, smoking

## Abstract

**Background and purpose:**

We aimed to investigate the effect of smoking on the risk of intracranial aneurysm (IA) rupture (IAR), specifically relationship between the number of cigarettes smoked per day (CPD) or smoking index and the risk of IAR.

**Methods:**

We performed a single-center case–control study of consecutive patients evaluated or treated for IA at our institution from June 2015 to July 2016. Cases were patients with a ruptured IA. Two age- and sex-matched controls with an unruptured IA were included per case. Conditional logistic regression models were used to assess the relationship between both the CPD and smoking index (CPD × years of smoking) and IAR.

**Results:**

The study population included 127 cases of IAR and 254 controls. The higher IAR risk was associated with cigarette smoking (both current and former) (OR, 2.3; 95% CI, 1.1–4.8; *P* = 0.029). Our subgroup analysis of smokers revealed a significant association between IAR risk and current smoking (OR, 2.8; 95% CI, 1.2–6.3; *P* = 0.012), current heavy smoking (CPD ≥ 20) (OR, 3.9; 95% CI, 1.4–11.0; *P* = 0.007), and a smoking index ≥800 (OR, 11.4; 95% CI, 2.3–24.5; *P* = 0.003). Former smoking was not significantly associated with IAR (OR, 1.1; 95% CI, 0.3–4.0; *P* = 0.929).

**Conclusion:**

A dose–response relationship has been noted for intensity and duration of smoking consumption and increased risk of IAR. As smoking is modifiable, this finding is important to managing patients with IAs to quit or reduce smoking prior to life-threatening subarachnoid hemorrhage.

## Introduction

Nearly 3% of the adult population was found to have an unruptured intracranial aneurysm (IA) (1). With the increasing use of cranial imaging, more incidental IAs is being detected in clinical practice. Although the annual risk of rupture of asymptomatic IA is relatively low, subarachnoid hemorrhage (SAH) caused by IA rupture associated with high rates of morbidity and mortality (2). Prevention is better than cure. Hence, to prevent SAH *via* modifiable risk factors identification and management, rather than surgical clipping and intravascular intervention, is of great clinical and social value.

Smoking is the most important established risk factor for IA rupture (3–6), and up to 80% of patients who sustain an aneurysmal SAH have a history of smoking, and 50–60% are current smokers (7, 8). A Finnish register-based study reported that the incidence of SAH was decreasing and this trend may be associated with changes in smoking rates, suggesting the possible benefits of smoking cessation (9). However, the predictors of adverse outcome after smoking exposure have not been clearly identified, and most studies have simply stratified subjects based on the presence or absence of a history of cigarette smoking (7, 8).

Using quantitative indicators within the smoking history, such as the number of cigarettes smoked per day (CPD) and smoking index (CPD × years of tobacco use) (10), may lead to a more detailed understanding of the mechanisms by which smoking contributes to aneurysm formation and rupture and helps to develop strategies to reduce the risk of rupture. We, therefore, devised a case–control study to investigate the effect of smoking on the risk of IA rupture, specifically the relationship between the CPD and smoking index and rupture risk.

## Materials and Methods

### Patient Selection

We performed a single-center case–control study of consecutive patients evaluated or treated for IA at our institution from June 2015 to July 2016. Cases were defined as patients with SAH secondary to a ruptured IA and controls as patients harboring a UIA. Two controls were randomly selected from the patients admitted with a diagnosis of UIA and matched to each case based on age (±5 years) and sex. All enrolled patients were examined using three-dimensional rotational angiography. For all cases, SAH was diagnosed using computed tomography (CT).

Our exclusion criteria included: (1) dissecting, fusiform, traumatic, mycotic, or partially thrombosed aneurysms; (2) the patients with nonaneurysmal SAH examinated by digital subtraction angiography (DSA), or the location of ruptured aneurysm could not be identified among multiple IAs by CT and DSA; (3) aneurysms without clear and readable three-dimensional rotational angiography that allowed an evaluation of lesion geometry and morphology; (4) aneurysms associated with cerebral arteriovenous malformation, arteriovenous fistula, or moyamoya disease.

The study was approved by the review committee of our hospital, and informed consent was obtained from all the subjects.

### Data Collection and Definitions

Information on smoking was obtained from the medical history recorded by the treating physicians during interviews of patients or family members. If the patient’s information was incomplete, we obtained information using a telephone survey. All patients were asked “Have you ever smoked?” and if the answer was yes, “For how many years in total have you smoked?”, “how long ago did you quit smoking?”, and “Do you currently smoke?” Individuals who were current smokers or former smokers were asked to report the mean number of cigarettes, pipes, and cigars smoked per day. Nonsmokers affirmed that they had never smoked or smoked <100 cigarettes (lifetime). Patients who smoked at the time of treatment or smoked ≥100 cigarettes during the past year were considered current smokers. Patients who had smoked ≥100 cigarettes but had not smoked during the past year were considered former smokers (10).

The smoking index is a unit for measuring cigarettes consumption over a long period and was calculated using the following formula: smoking index = CPD × years of tobacco use. Smoking index categories were nonsmoker, <400, 400–799, and ≥800 (11). The CPD was estimated for current and former smokers. We defined heavy smoking as ≥20 CPD and mild smoking as <20 CPD (12).

We investigated other potential risk factors for aneurismal rupture. Social-demographic characteristics included age, sex, and educational level, and clinical characteristics included body mass index (BMI), comorbidities, coronary artery procedures, alcohol use (current or previous intake >5 drinks per day), and family history of IA. A BMI ≥25 kg/m^2^was defined as overweight (13).

We measured the aneurysm dimensions, neck size (mm), parent artery diameter (mm), aspect and size ratios, shape, and location. The aneurysm size and height were defined as the largest cross-sectional diameter and the maximum perpendicular distance between the neck and any point on the aneurysm dome, respectively. The aneurysm width was defined as its maximum horizontal length. The aneurysm height/width ratio was also calculated. The aspect ratio was defined as the height of the aneurysm compared to its average neck size. Bifurcation location was considered as aneurysms located at parent artery bifurcations in the circle of Willis, and, therefore, originated from more than one parent vessel (internal carotid artery terminus, anterior communicating artery, internal carotid-posterior communicating artery, middle cerebral artery bifurcation, and apex of the basilar artery) (14). The size ratio was defined as the aneurysm height compared to the mean vessel diameter of all vessel branches associated with the aneurysm (15). The vessel diameter was calculated as the average of the vessel diameter at the neck of the aneurysm (D1) and the diameter of the cross section located 1.5 × D1 mm from the aneurysm neck.

All morphological parameters were obtained by three-dimensional rotational angiography and evaluated by two experienced neurosurgeons.

### Statistical Analyses

All statistical analyses were performed using SPSS Statistics for Windows (Version 22.0; IBM Corp., Armonk, New York, USA). Continuous variables were analyzed using the Mann–Whitney *U* test or Student’s *t*-test and are presented as mean ± SD or medians (interquartile ranges). Categorical variables were analyzed using Fisher’s exact test or the Pearson chi-square test and are presented as frequencies (percentages). Associations between smoking history and intracranial aneurysm rupture (IAR) were assessed using Fisher’s exact test or the linear-by-linear association test. Because our study was performed on a matched sample, the Cochran–Mantel–Haenszel test was also performed. As predetermined, variables with a *P* < 0.20 in the univariate logistic regression analysis were evaluated in our multivariate analysis. Because the sample was matched, we used conditional logistic regression to calculate univariate and multivariate odds ratios (ORs) with 95% confidence intervals (CI). A *P*-value <0.05 was regarded as statistically significant.

## Results

**Figure 1:**
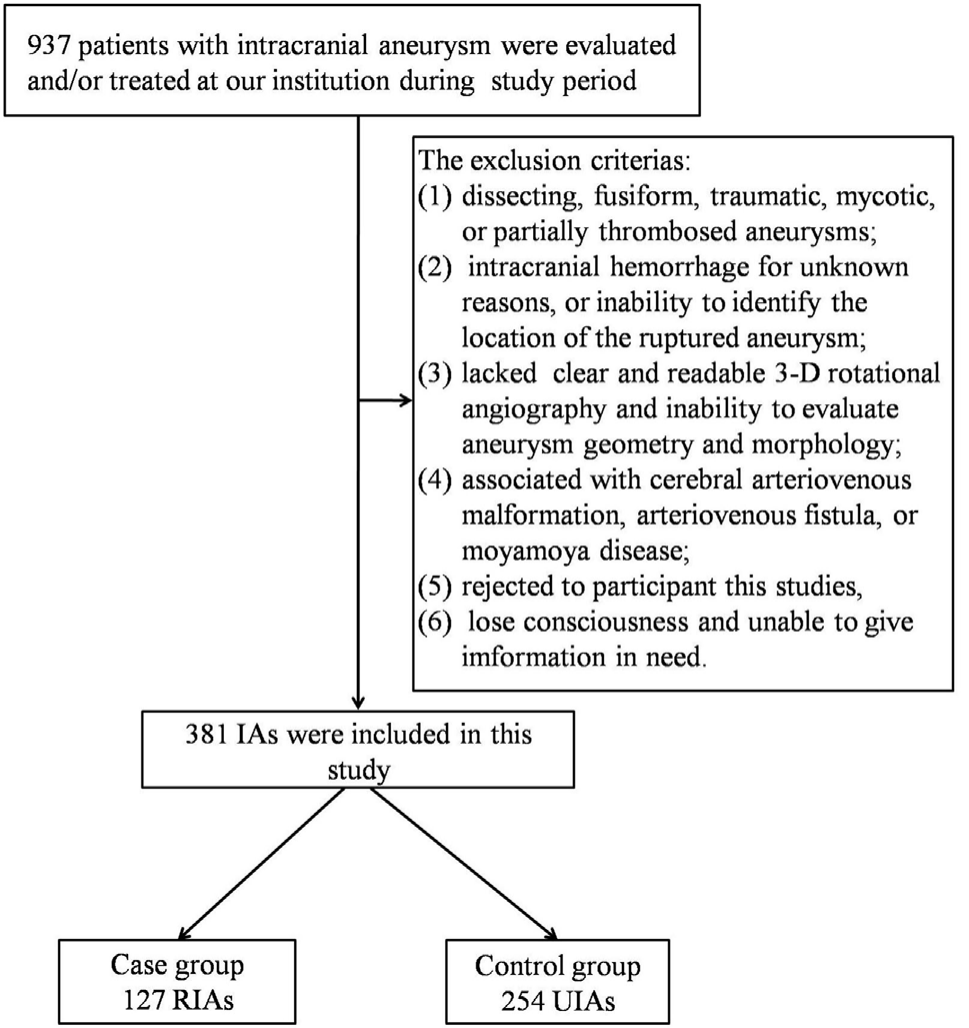
Study Flowchart.

A total of 937 patients with IAs were evaluated or treated at our institution during the study period. After applying our exclusion criteria, 147 case of IAR and 526 cases of unruptured IAs were included. However, 20 cases of ruptured IAs failed to find their matched controls. Consequently, our study population was composed of 127 cases of IAR and 254 matched controls with UIAs (Figure 1). Table 1 shows the distribution of demographic and clinical characteristics in both groups.

**Table 1:** Distribution of age, sex, and locations of cases and controls.

Characteristics	Cases	Controls
Number	127	254
Female, *n* (%)	74 (58.3%)	148 (58.3%)
Male, *n* (%)	53 (41.7%)	106 (41.7%)
**Age (years)**		
Mean ± SD	54.0 ± 10.9	54.6 ± 9.8
20–29	4 (3.1%)	8 (3.1%)
30–39	7 (5.5%)	14 (5.5%)
40–49	30 (23.6%)	60 (23.6%)
50–59	43 (33.9%)	86 (33.9%)
60–69	35 (27.6%)	70 (27.6%)
≥70	8 (6.3%)	16 (6.3%)
**Locations of aneurysms, ***n***(%)**		
ICA	13 (10.2%)	133 (52.4%)
ACOM	42 (33.1%)	25 (9.8%)
PCOM	31 (24.4%)	31 (12.2)
MCA	2 (1.6%)	10 (3.9%)
ACA	33 (21.3%)	27 (13.0%)
PC	20 (9.4%)	12 (8.7%)

*PCOM, internal carotid-posterior communicating artery; ACOM, anterior communicating artery; MCA, middle cerebral artery; ICA, internal carotid artery; PC, basilar tip and basilar-superior cerebellar artery and vertebral artery-posterior inferior cerebellar artery and vertebrobasilar junction; ACA, anterior cerebral artery*.

Table 2 shows the frequency and odds of risk factors in IAR cases compared to controls. The following covariates met our previously determined level of significance and entered the stepwise forward selection for the conditional logistic model: smoking status (nonsmoking, former smoking, and current smoking; *P* = 0.013), BMI ≥ 25 (*P* = 0.168), alcohol use (*P* = 0.012), history of hyperlipidemia (*P* = 0.012), diabetes mellitus (*P* = 0.112), aneurysm size (*P* = 0.192), and height/width ratio (*P* = 0.097). Cases and controls also showed significant differences in frequency and risk of IAR based on smoking status determined by CPD [nonsmoker, former smoker, mild current smoking (CPD <20), and heavy current smoking (CPD ≥20); *P* = 0.02] and smoking index (nonsmoker, former smoker, and current smoker with smoking indexes of <400, 400–799, and ≥800, respectively; *P* = 0.017).

**Table 2:** Frequency and odds of vascular risk factors in cases compared to controls.

Characteristics	Cases	Controls	*P*-value	OR (95% CI)
Number	127	254		
Educational level				
University or more	34 (26.8%)	63 (24.8%)	0.678	1.1 (0.7–1.8)
Characteristics of smokers				
Smoking status				
Nonsmoker	77 (60.6%)	186 (73.2%)	Reference	Reference
Former smokers	7 (5.5%)	17 (6.7%)	0.564	1.4 (0.5–4.1)
Current smokers	43 (33.9%)	51 (20.1%)	0.001	3.3 (1.6–6.5)
Smoking index, *n* (%)			0.017	
Nonsmoker	77 (60.6%)	186 (73.2%)	Reference	Reference
Former smokers	7 (5.5%)	17 (6.7%)	0.532	1.4 (0.4–4.3)
Current smoking				
400<	17(13.4%)	23 (9.1%)	0.012	3.0 (1.3–7.2)
400–799	9 (7.1%)	16 (6.3%)	0.265	1.8 (0.6–4.9)
>800	17 (13.4%)	12 (4.7%)	<0.001	7.2 (2.4–21.6)
Number of cigarettes per day			0.021	
Nonsmoker	77 (60.6%)	186 (73.2%)	Reference	Reference
Former smokers	7 (5.5%)	17 (6.7%)	0.536	1.4 (0.5–4.2)
Current smoking				
<20 per day	12 (9.4%)	19 (7.5%)	0.115	1.9 (0.9–4.4)
≥20 per day	31 (24.2%)	32 (12.6%)	0.001	4.0 (1.8–8.9)
Duration of smoking, (mean ± SD)^a^	25.3 ± 11.3	23.3 ± 9.6	0.297	
Alcohol use, *n* (%)	47 (18.5%)	38 (29.9%)	0.012	1.9 (1.1–3.1)
Overweight(BMI ≥ 25)	55 (43.3%)	129 (50.8%)	0.168	0.7 (0.5–1.1)
Hypertension, *n* (%)	68 (58.5%)	133 (52.4%)	0.828	1.0 (0.7–1.6)
Hyperlipidemia, *n* (%)	11 (8.7%)	47 (18.5%)	0.012	0.4 (0.2–0.8)
Diabetes mellitus, *n* (%)	8 (6.3%)	29 (11.4%)	0.112	0.5 (0.2–1.2)
Cerebral ischemic comorbidities, *n* (%)	12 (9.4%)	29 (11.4%)	0.559	0.8 (0.4–1.6)
Cardiac comorbidities, *n* (%)	6 (4.7%)	16 (6.3%)	0.534	0.7 (0.3–1.9)
Previous SAH, *n* (%)	4 (3.1%)	6 (2.4%)	0.737	1.3 (0.4–4.8)
Multiplicity, *n* (%)	28 (22.0%)	56 (22.0%)	1.000	1.0 (0.6–1.7)
Aneurysm size ≥ 5 mm	72 (56.7)	125 (49.6)	0.192	1.3 (0.9–2.0)
Irregular shape	72 (56.7%)	131 (51.6%)	0.345	1.2 (0.8–1.9)
Aspect ratio	47 (37.0%)	81 (31.9%)	0.319	1.3 (0.8–2.0)
Size ratio	43 (33.9%)	80 (31.2%)	0.642	1.1 (0.7–1.8)
Height/width ratio	53 (41.7%)	84 (33.7%)	0.097	1.4 (0.9–2.2)
Flow angle ≥ 90	98 (77.2%)	174 (69.3%)	0.109	1.4 (0.9–2.5)
Bifurcation, *n* (%)	93 (73.2%)	74 (29.1%)	<0.001	6.7 (4.1–10.7)
Location of PC, *n* (%)	20 (9.4%)	12 (8.7%)	0.799	1.1 (0.5–2.3)

*^a^Among current and former smokers*.

*BMI, body mass index; CI, confidence interval; PC, basilar tip and basilar-superior cerebellar artery and vertebral artery-posterior inferior cerebellar artery and vertebrobasilar junction; OR, odds ratio; SAH, subarachnoid hemorrhage*.

*Odds ratios show results of Cochran–Mantel–Haenszel test for cases Vs controls or unadjusted conditional logistic regression results. The P-values are results of Pearson chi-square tests*.

### The Association Between Smoking and IAR

**Figure 2:**
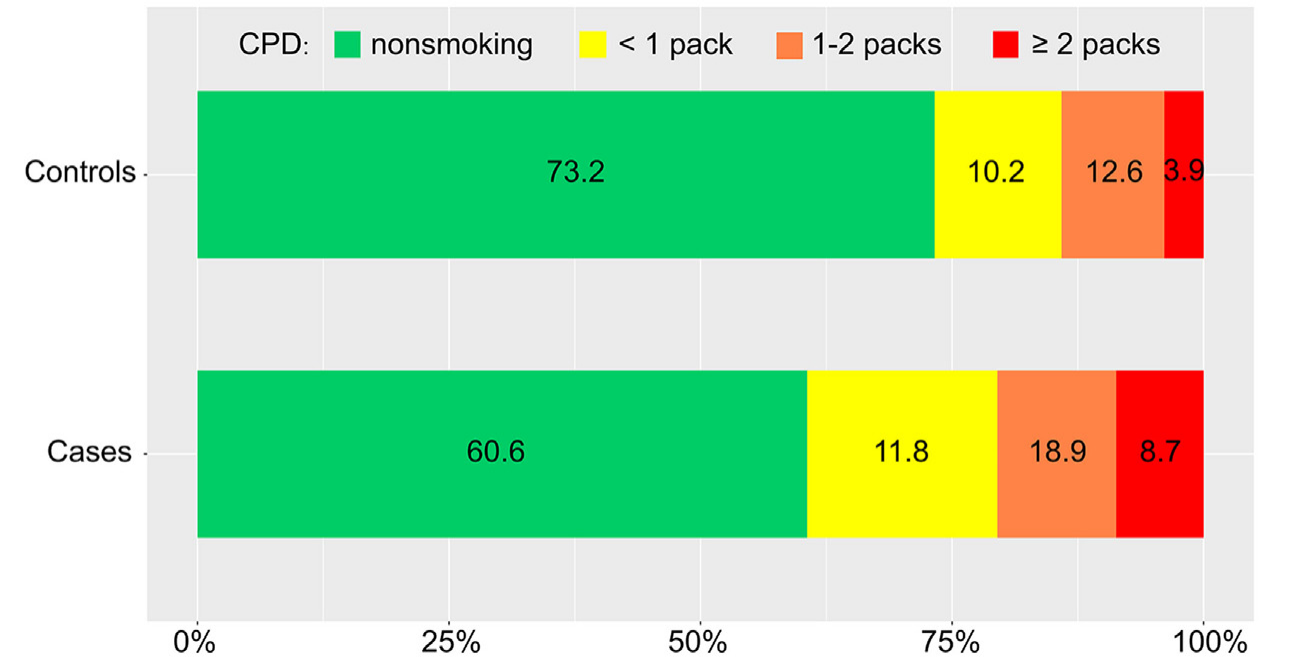
Distribution of daily cigarette intake in cases and controls. CPD, number of cigarettes smoked per day.

**Figure 3:**
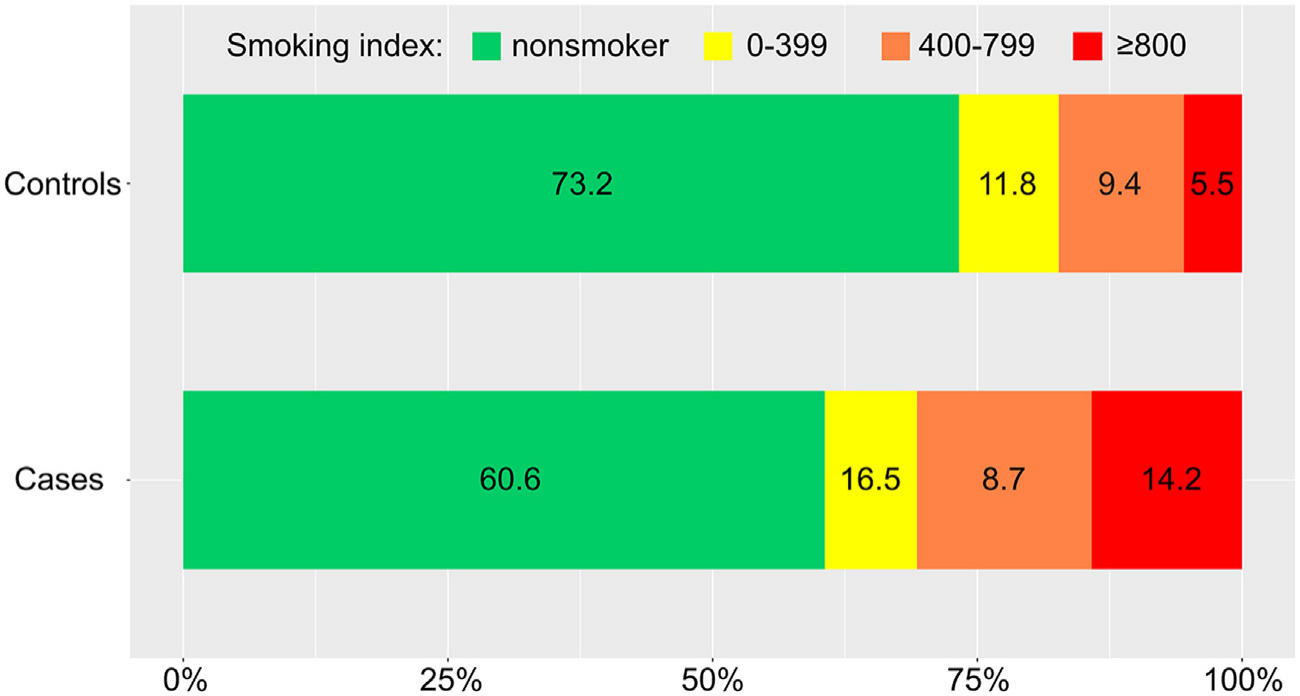
Distribution of smoking index (number of cigarettes smoked per day × years of smoking) in cases and controls.

Distributions of the CPD and smoking index in cases and controls were showed in Figures 2 and 3 (*P* = 0.019, *P* = 0.009, respectively). The results of our conditional logistic regression analysis of characteristics related to IAR are shown in Table 3. After adjusting for BMI, alcohol use, history of hyperlipidemia, history of diabetes mellitus, and aneurysm aspect and height/width ratios, cigarette smokers (current and former) had a significantly increased risk of SAH compared with nonsmokers (OR, 2.3; 95% CI, 1.1–4.8; *P* = 0.029). Besides, we respectively entered smoking statuses (nonsmoking/current smoking/former smoking), CPDs, and smoking indexes into the conditional logistic regression model. The risk was twice as high in current smokers (OR, 2.8; 95% CI, 1.2–6.3; *P* = 0.012) compared to former smokers (OR, 1.0; 95% CI, 0.3–4.0; *P* = 0.929), although the association between former smoking and IAR was not statistically significant. Current heavy smoking (CPD ≥20) was significantly associated with IAR (OR, 3.9; 95% CI, 1.4–10.9; *P* = 0.007), although the same was not true of current smoking with a CPD ≤20 (OR, 1.8; 95% CI, 0.6–5.3; *P* = 0.292). The ORs for a current smoking index of less than 400 and 400–799 were relative to 800 or higher. The point estimates for the ORs were 11.4 (smoking index ≥800; 95% CI, 2.3–24.5; *P* = 0.003).

**Table 3:** Conditional logistic regression analysis related to intracranial aneurysm rupture.

Characteristics	OR (95% CI)	*P-*value
Bifurcation aneurysm	11.0 (5.4–22.1)	<0.001
Overweight [body mass index (BMI) ≥25]	0.6 (0.3–1.0)	0.045
Diabetes mellitus	0.3 (0.1–0.9)	0.032
Hyperlipidemia	0.3 (0.1–0.8)	0.016
Smoking (current/former smoking)	2.3 (1.1–4.8)	0.029
Conditional logistic regression results for current/former smoking^a^		
Nonsmoker	Reference	
Vs former smoker	1.1 (0.3–4.0)	0.929
Vs current smoker	2.8 (1.249–6.303)	0.012
Conditional logistic regression results for current mild/heavy smoking^a^		
Nonsmoker	Reference	
Vs former smoking	1.1 (0.2–4.2)	0.886
Vs current mild smoking	1.8 (0.6–5.3)	0.292
Vs current heavy smoking	4.0 (1.4–11.0)	0.007
Conditional logistic regression results for current smoking with different smoking index^a^		
Nonsmoker	Reference
Vs former smoker	1.0 (0.3–4.0)	0.989
Vs current smoking index <400	2.7 (0.9–8.2)	0.077
Vs current smoking index ≥400 and <800	1.4 (0.4–4.7)	0.686
Vs current smoking index ≥800	11.4 (2.3–24.5)	0.003

*^a^Adjusting for history of diabetes, history of hypertension, frequent alcohol use, education, and BMI*.

*OR, odds ratio. Heavy smoking, ≥20 per day; mild smoking, <20 per day*.

### Other Risk Factors

In the conditional logistic regression models, bifurcation location significantly increased the risk of IAR (OR, 11.0; 95% CI, 5.4–22.1; *P* < 0.001). The risk of IAR was lower in overweight patients (OR, 0.6; 95% CI, 0.3–1.0; *P* = 0.045) and those with hyperlipidemia (OR, 0.3; 95% CI, 0.1–0.8; *P* = 0.016), or diabetes mellitus (OR, 0.3; 95% CI, 0.1–0.9; *P* = 0.032). What is more, we analyzed the risk for IAR of bifurcation location among smokers (current smoking/former smoker) and nonsmokers, respectively. The results were showed in Table 3.

## Discussion

In this case–control study, we demonstrated that a CPD ≥20 and a high smoking index were strong risk factors for IAR. Furthermore, compared with former smokers who had quit at least 1 year before evaluation, current smokers were more predisposed to IAR, suggesting the importance of smoking cessation in patients with an IA.

Similarly, in their case–control study of 250 patients with an aneurysmal SAH and 206 patients with a UIA, Monique et al. found that current smoking increased the risk of IAR (7). One possible explanation is that most smoking-induced changes are reversible after quitting, although previous studies suggested that former smokers demonstrate an ongoing low-grade inflammatory response that persists long after smoking cessation (14). Cigarette smoke is an aerosol containing thousands of chemicals, including nicotine, carbon monoxide, and oxidant compounds (14), and chronic exposure induces multiple pathological effects in the vascular endothelium and facilitates inflammation. This process consistently weakens the UIA wall making it more vulnerable to trigger factors and eventually leads to rupture. Aneurysm growth has also been shown to be increased by current smoking supporting the concept that smoking increases SAH particularly by increasing aneurysm size and possibility of rupture (16, 17).

However, some authors have proposed that smoking may not be a trigger for aneurismal rupture (18). These contradicting results are probably explained by the lack of standard in design in which no details on the amount of cigarettes consumed were available. As we asked a detailed question with regards to the CPD, we were able to assess the precise consumption, which is strength of this study. In our study, we found that increased CPD was associated with higher percentages of IAR in cases Vs controls (Figure 2). Furthermore, in conditional logistics model, current heavy smoking (CPD ≥20) was significantly associated with IAR with a relative risk of 3.9, while current light smoking was not consistent with this finding; Juvela et al. assessed smoking intensity defined as the mean number of CPD and found that patients with aneurysmal SAH had heavier tobacco use than those without (19). In smokers, an accumulation of smoke-induced chemical mediators has been positively correlated within creased impairment of the endothelial capacity and, therefore, an increased risk of IAR (20).

Although the CPD was a strong and independent risk factor for UIAs in our study, it should be noted that this straightforward index of cumulative exposure does not take the duration of smoking into account. Therefore, we used the smoking index to investigate whether the increased risk of IAR due to smoking is modifiable. This index can make strong assumptions regarding the equivalence of the roles of intensity and duration (21). We found a significant association between the smoking index and the risk of IAR (Figure 3). Our results are congruent with the basic research findings that the effects of cigarette smoke on endothelial cells are only functional initially, while the endothelial-cell layer exhibits physical damage and can even be completely destroyed by chronic exposure of cigarette smoke (increase of smoking index) (22). Moreover, increasing degrees of smoking may be more permissive to accelerate morphological changes of aneurysms (23). These changes, in turn, may increase the eventual rupture risk of the aneurysm. We confirmed this association using a conditional logistic regression model. However, the association between a smoking index of 400–799 and the risk of IAR did not reach statistical significance in our study. This result may be due to our retrospective study design and limited sample size.

In our study, 73.2% of ruptured aneurysms were in a bifurcation location, and this location was an independent risk factor that raised the risk of IAR by a factor of 10.972. This finding is consistent with previous reports (21). The arterial wall is consistently weakened, where it bifurcates because this area correlates with increased hemodynamic stress and higher blood flow. Thus, IAs in bifurcation locations have a higher risk of rupture than those in other locations (24). What is more, we analyzed the risk for IAR of bifurcation location among smokers (current smoking/former smoker) and nonsmokers, respectively. Our results showed that the bifurcation risk increased for smokers and nonsmokers.

The risk of IAR in female smokers might be greater than the rupture risk in male smokers. This hypothesis is supported by several studies reporting a dramatic sex-based difference in risk at a given level of cigarette consumption (24, 25). In their community-based case–control analysis, Bonita et al. demonstrated that cigarette smokers had a significantly increased risk of SAH compared with nonsmokers with relative risks of 3.0 and 4.7 for men and women, respectively (24). Age is an important independent risk factor for acute aneurysm rupture (18, 25), and one of the most important factors affecting cigarette consumption duration. We matched cases based on year of birth (age ± 5 years) and sex to avoid introducing variability.

Our study showed that hyperlipidemia independently decreased the risk of IAR. Recently, several studies reported similar results (5, 21). Interestingly, a recent study reported an association between the administration of statins and reduced UIA formation in rats (21). It may be that hypercholesterolemic patients treated with statins gain a similar protective benefit. BMI also independently decreased the risk of IAR, a finding similar to that reported by Monique et al. (5). In their study of 305 patients with SAH, Hughes et al. reported that BMI was inversely related to short- and long-term mortality (26). However, few other studies demonstrating an association between BMI and aneurysm rupture are available. We also found that diabetes mellitus was associated with a decreased risk of IAR. It has been hypothesized that many patients had presented for evaluation of their diabetes mellitus, allowing some IAs to be diagnosed before they ruptured or altering lifestyle factors and continuing medical care to reduce the risk of SAH. However, the biological foundation of this inverse correlation needs further investigation.

It should be noted that various factors, such as previous SAH, hypertension, location in the posterior circulation, larger AR, and larger size have been reported to increase the risk of IAR (27–29). However, in this study, these factors were not significantly related to rupture. These inconsistencies might be attributed to the instability of the factors, limited sample of our study and design of matched cases. Although the case group included more patients with at least an education level of high school than the control group (54.4 Vs 48.8%, respectively), the level of education was not significantly associated with IAR in this study.

### Strengths and Limitations

Our study has several strengths. First, this study has avoided concerns of previous retrospective studies by use of sex- and age-matched controls for patients with ruptured IAs. As all study patients came from the same hospital within 2 years, we were able to perform our analysis in a defined population. Second, the rigorous measurement of aneurysm characteristics by two experienced neurosurgeons ensured a representative and fair comparison between cases and controls. Finally, to minimize recall bias, we included only patients evaluated or treated at our institution within 2 years of our study start date.

However, the study had several limitations. First, it was a retrospective study from a single center, suggesting that the collection of data was potentially biased. Second, the aneurysm features, such as size, AR, and SR, might change after rupture, and these changes could cause bias. Finally, we did not take passive smoking into account, because it is difficult to determine the intensity and duration of passive smoking, and most smokers were also passive smokers. Further study of the effects of passive smoking on IAs is needed.

## Conclusion

Patients with IA who smoked had a greater risk of aneurysm rupture compared with those who were nonsmokers or former smokers. The strength of the association between current smoking and the risk of aneurysmal rupture was directly related to the smoking index and CPD. Information from the current study may be beneficial to increase the potential for patients harboring IAs to quit or reduce smoking prior to life-threatening SAH.

## Data Sharing Statement

The authors agree to share any data on request. Any data from this study are available by contacting the corresponding author.

## Author Contributions

Conceived and designed the study: AL. Performed the study: XF, LW, EG and BZ. Analyzed the data: XF and ZQ. Contributed reagents/materials/analysis tools: ZQ, PL, XW, WX, YL, and CJ. Wrote the paper: XF and ZQ. Revised the manuscript: ZW.

## Conflict of Interest Statement

The authors declare that the research was conducted in the absence of any commercial or financial relationships that could be construed as a potential conflict of interest.
